# Supplementary material for: Geographic context affects the landscape change and fragmentation caused by wind energy facilities
Source: PeerJ. 2019 Jul 16;7:e7129. doi: 10.7717/peerj.7129 (PMC6640624; doi:10.7717/peerj.7129)

Supplemental materials: Maps of 5 wind facilities pre and post construction

Figure 1. Goodnoe Wind Facility

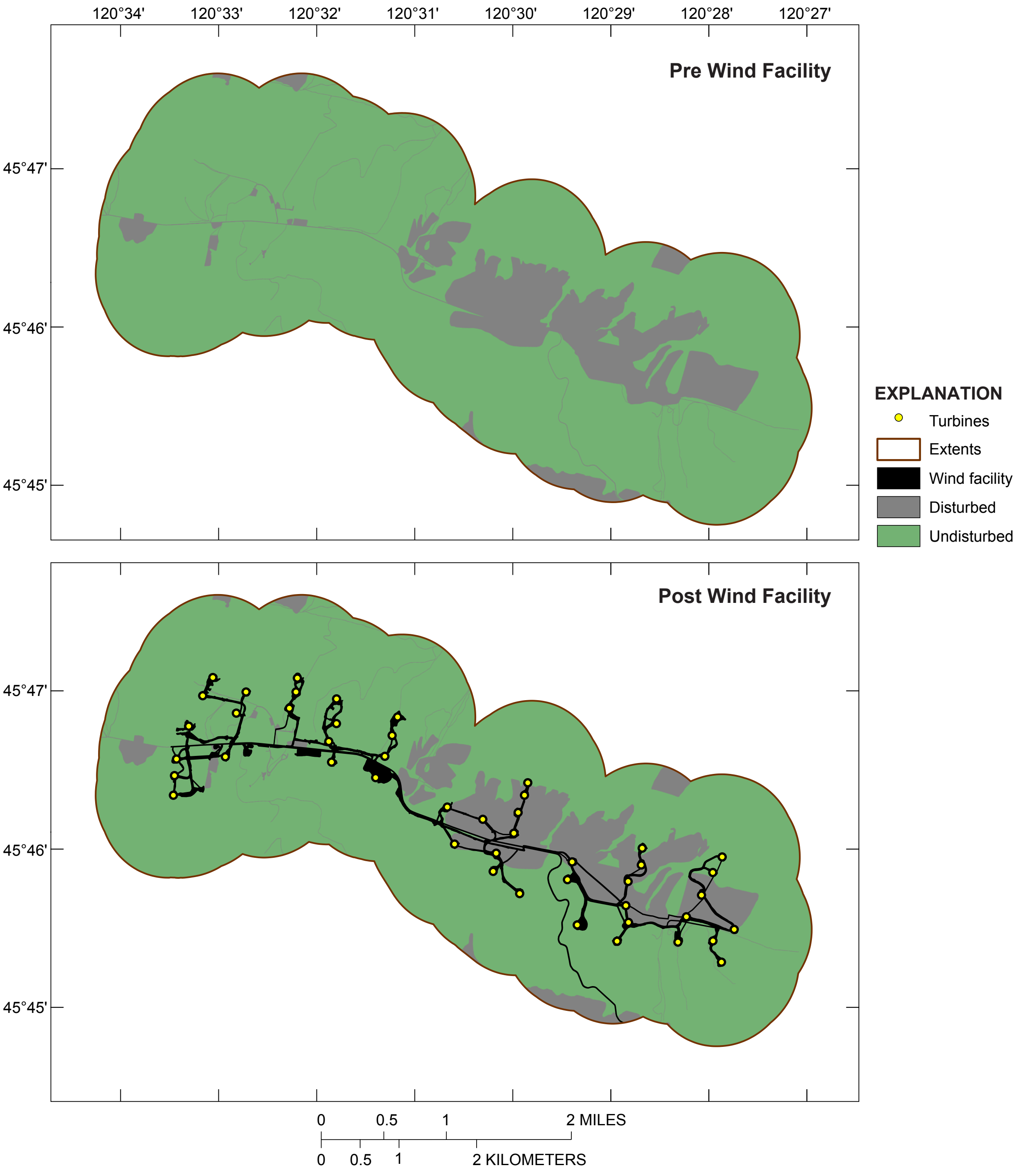

Figure 2. Cerro Gordo Wind Facility

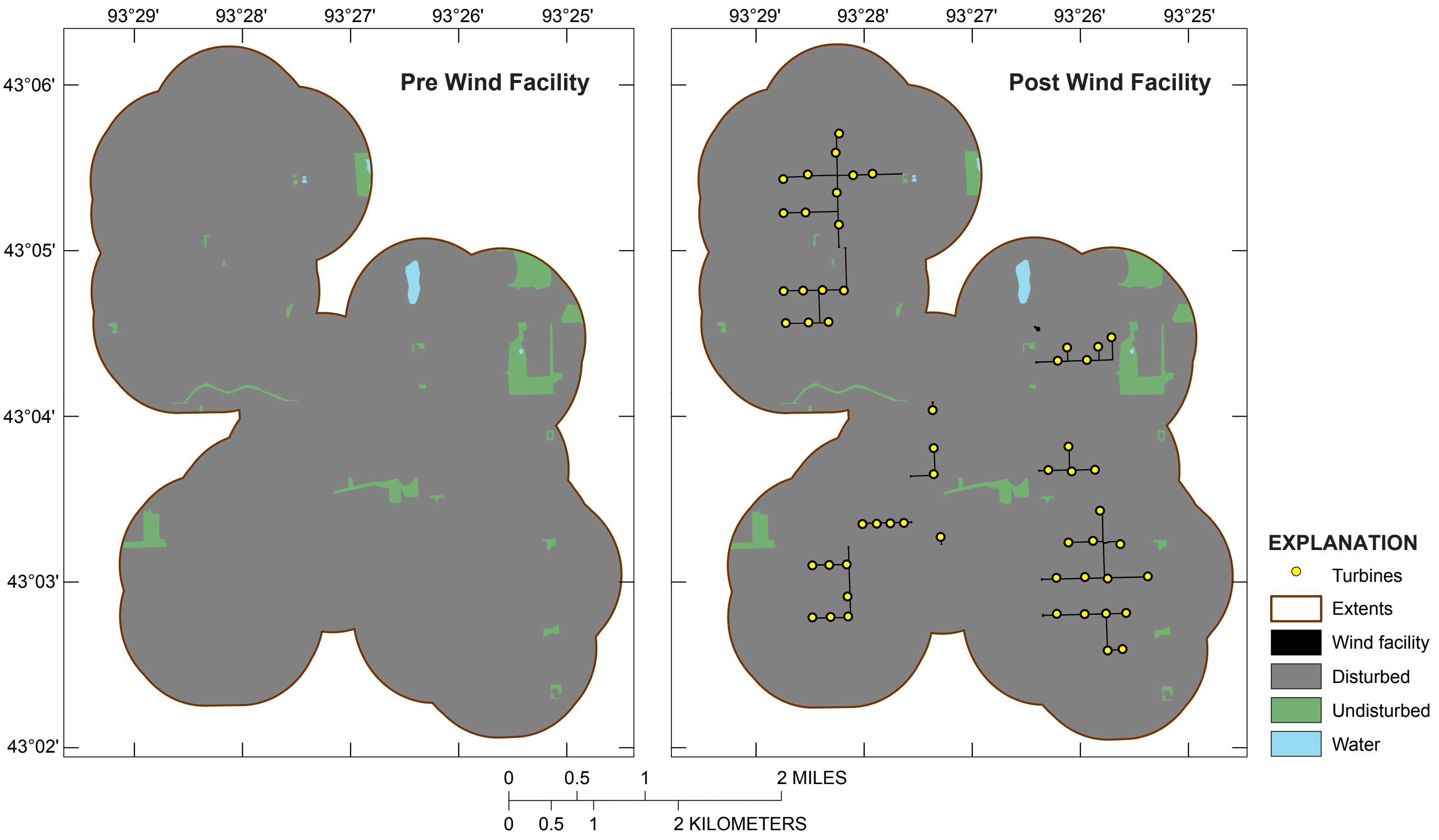

Figure 3. Foote Creek Wind Facility

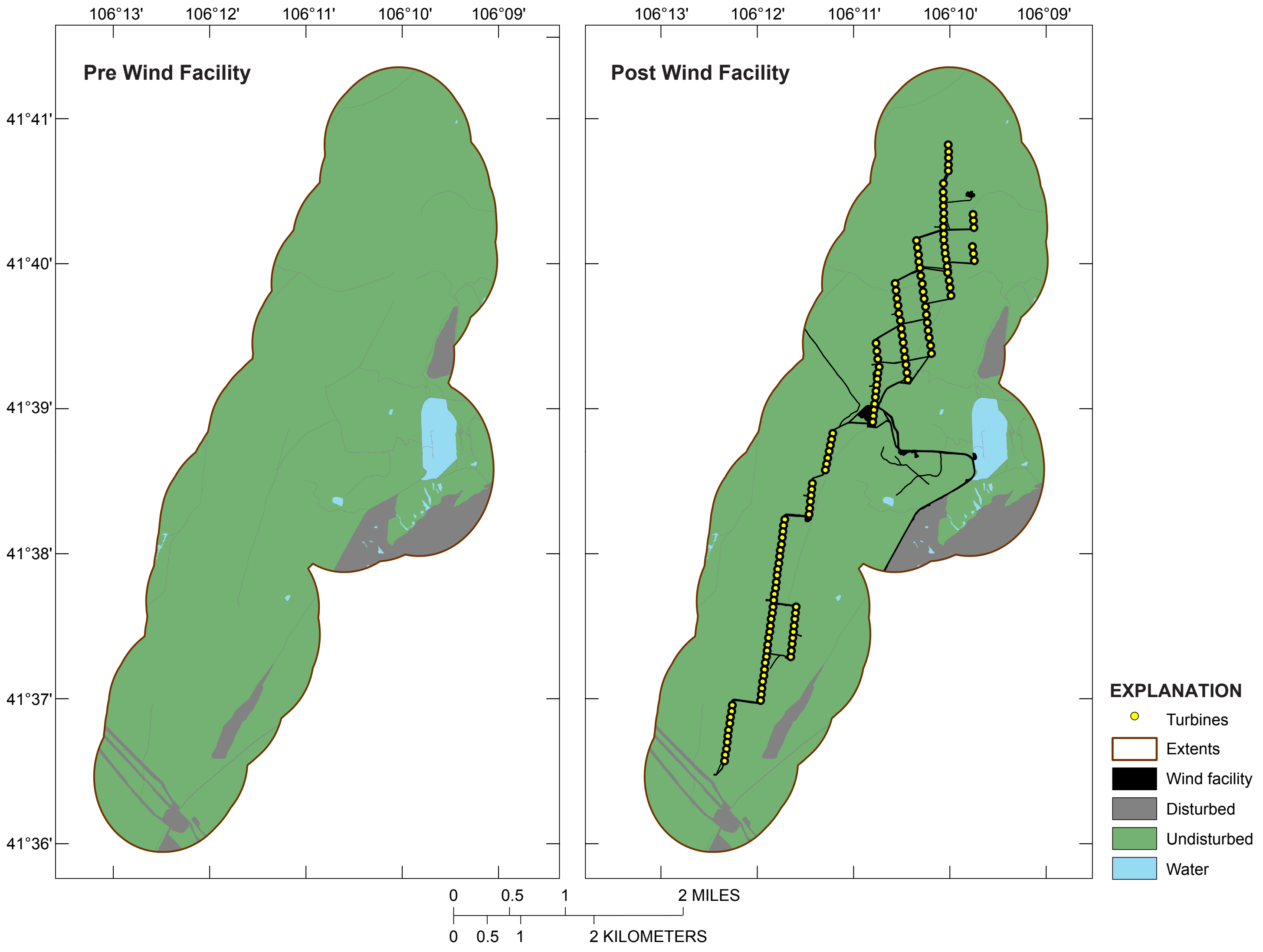

Figure 4. White Creek Wind Facility

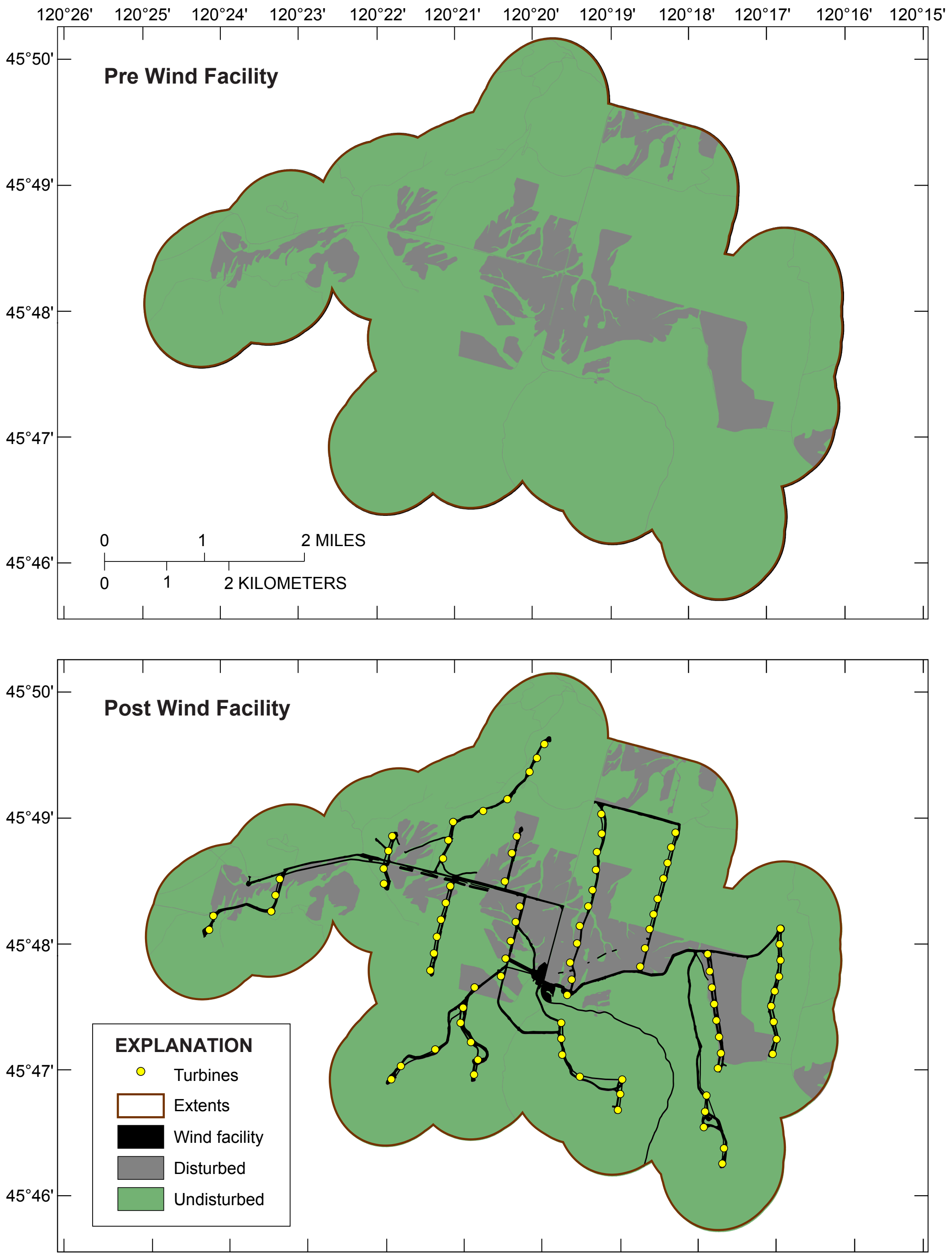

Figure 5. Oliver Wind Facility

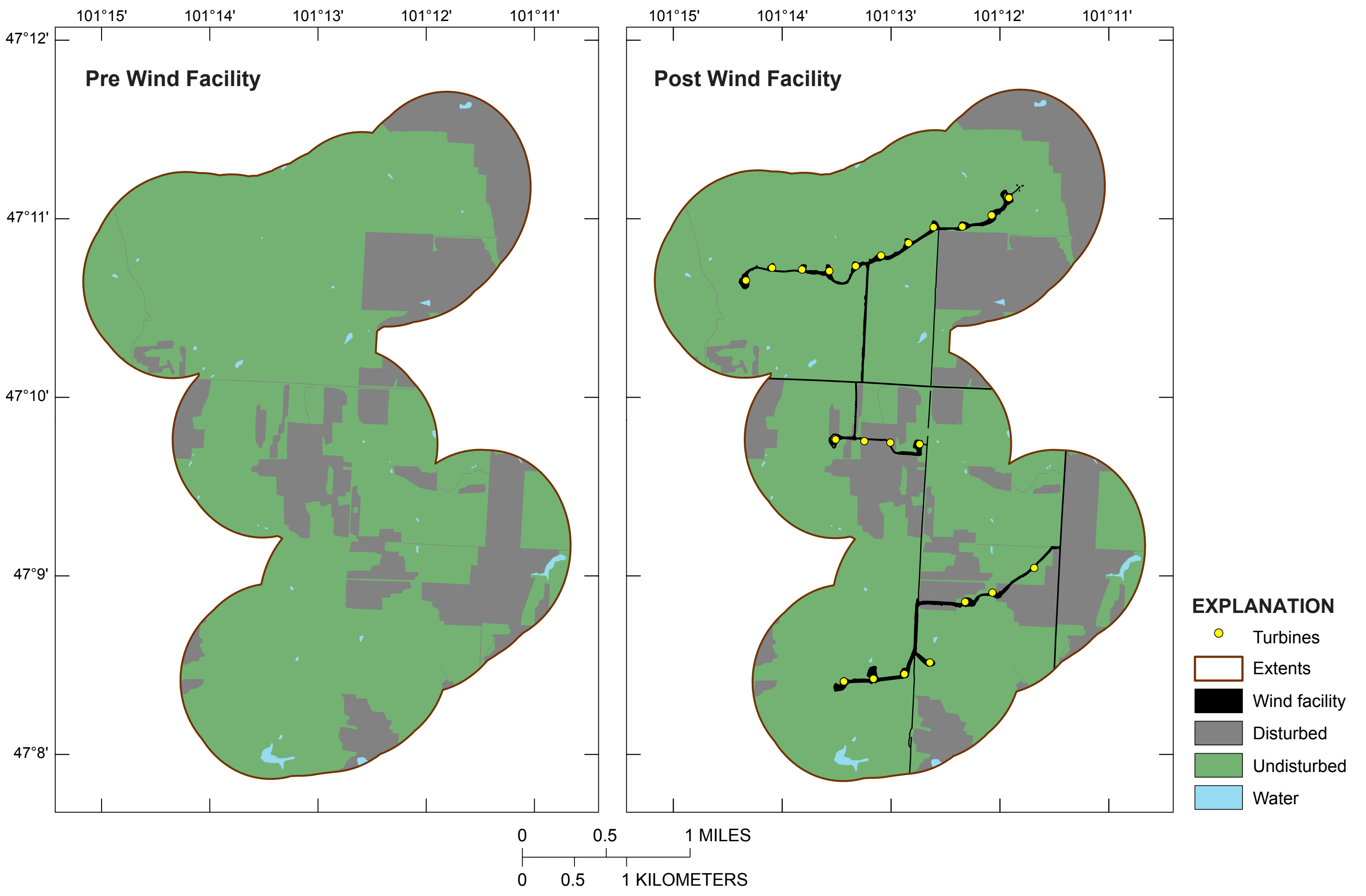

Supplement: Supplemental Information 1 [file peerj-07-7129-s001.pdf]
